# Micro‐Supercapacitors for Self‐Powered Biosensors

**DOI:** 10.1002/smsc.202400096

**Published:** 2024-05-28

**Authors:** Muhammad Adeel, Hong Seok Lee, Kanwal Asif, Sabrina Smith, Hasan Kurt, Flavio Rizzolio, Salvatore Daniele, Firat Güder

**Affiliations:** ^1^ Department of Bioengineering Royal School of Mines Imperial College London London SW7 2AZ UK; ^2^ Department of Molecular Sciences and Nanosystems Ca’Foscari University of Venice 30123 Venezia Italy

**Keywords:** biosensing, energy harvesting, microsupercapacitors, self‐powered sensors, wearable biosensors

## Abstract

Although batteries are a highly popular energy source in biosensors, batteries can be an economic limitation for low‐cost sensing applications and pose significant challenges in miniaturization, biocompatibility, and disposal. To surmount such issues, “self‐powered sensors” gain the spotlight thanks to their energy harvesting from the environment through embedded miniaturized systems. In this review, the recent developments in self‐powered devices are summarized with a specific focus on the integration of supercapacitors with sensors and biosensors. The working principles of microsupercapacitors, fabrication methods, their integration with biosensors, and their ultimate applications (i.e., biomedical monitoring and analytical biomarker detection) are described. Different energy harvesting systems are summarized and their integration with self‐powered sensors or biosensors is highlighted. The limitations and challenges of the existing approaches and the future of supercapacitor‐integrated sensing systems are also critically discussed.

## Graphical abstract

The review explores microsupercapacitor‐integrated sensors or biosensors, highlighting the drawbacks of traditional battery‐powered biosensors, e.g., cost, miniaturization, and disposal. This article focuses on the integration of supercapacitors with sensors and biosensors, outlining the principles, fabrication methods, and applications of microsupercapacitors. It also discusses various energy harvesting systems and their integration with self‐powered sensors, highlighting achievements and limitations in current approaches while envisioning future advancements in microsupercapacitor‐integrated sensing systems.

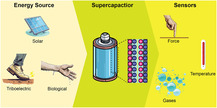

## 1 Introduction

Since the invention of microelectronic integrated circuits (ICs) in the 1950s, the density of transistors in ICs has been doubling about every 2 years, as described by Moore's law.^[^ ^1^ ^]^ Doubling the number of transistors in a unit die area enables more energy‐efficient operation and higher computational power from the similar size IC packaging.^[^ ^1^ ^]^ Unfortunately, Moore's law does not hold for batteries.^[^ ^1^ ^]^ Doubling the energy density of batteries can take more than decades; therefore, the battery technologies do not progress to the same extent as microelectronics or sensors in size, price, or performance. For most of the applications such as requiring, battery technologies continue to be the bottleneck for their development and adoption of wearable, implantable, or disposable biosensors technologies, which require portability, low cost, and miniaturization.

Biosensors are a specialized class of transducers that can convert the presence of biological, chemical, or biophysical entities into a detectable signal in a concentration‐dependent fashion.^[^ ^2^, ^3^, ^4^ ^]^ The detectable signals may include changes in electrical current/voltage, color, or other optical properties.^[^ ^3^, ^4^ ^]^ The choice of sensor or transduction method depends on various factors, such as sensitivity, selectivity, speed of execution of the measurements, ease of construction of the sensor, cost, and portability.^[^ ^3^, ^4^, ^5^, ^6^ ^]^ Each approach presents advantages and disadvantages, as documented in several reviews dealing with general aspects of sensors and their applications.^[^ ^3^, ^7^, ^8^ ^]^ Among other types, electrochemical sensors meet most of the above requirements.^[^ ^3^, ^4^, ^5^, ^6^ ^]^ Most sensor systems do not allow continuous monitoring due to several challenging issues, such as human–machine interface compatibility, heavy weight, and the need for external power sources to operate the devices continuously.^[^ ^9^, ^10^ ^]^ The latter requirement probably represents the most significant limitation to the continuous monitoring of body actions, which often forces the patient to resort to a hospital or clinic.^[^ ^10^, ^11^, ^12^ ^]^ Various energy storage and harvesting systems have been developed to enable continuous operation of sensors.^[^ ^13^, ^14^ ^]^ These technologies harness energy from external sources such as chemical reactions or solar power systems.^[^ ^14^, ^15^ ^]^ Energy storage systems, such as batteries, fuel cells, or supercapacitors (SCs), store modest amounts of energy and transform it into electrical signals.^[^ ^15^ ^]^ These signals are subsequently employed to provide continuous power to sensors, facilitating the uninterrupted monitoring of the targeted signals.^[^ ^15^ ^]^

Typically, batteries or capacitors are the main energy source for operating any portable electrical device, including sensors integrated into the human body and various other heavy machinery in daily life.^[^ ^10^, ^11^, ^12^, ^16^ ^]^ Batteries, however, have high energy density but low power density.^[^ ^11^ ^]^ On the other hand, capacitors store relatively low energy, but their power density is usually high. For wearable devices, energy storage and an energy supply unit are required, especially for the applicability of the device for long‐term continuous runs.^[^ ^7^, ^12^ ^]^ These characteristics can be provided by SCs, which have high power and energy densities, high storage capacity, and a long life cycle (up to 100 000 times).^[^ ^7^, ^16^ ^]^ They can be small in size, lightweight, and easy to manufacture. Miniaturized SCs can be integrated into chips and flexible substrates as energy‐storage microdevices. For these systems, the term micro‐SCs (μSCs) has been adopted.^[^ ^17^ ^]^ In wearable electronics or body‐integrated sensors, μSCs play an important role, as they can be integrated with sensors to produce microscale devices.^[^ ^7^, ^18^, ^19^, ^20^, ^21^ ^]^ Also, μSCs save and transform energy through various energy‐harvesting systems.^[^ ^7^, ^18^, ^21^ ^]^

The capacitive performance of the SCs is closely related to the nature and structure of the electrode materials, the electrolytes, and device designs. Novel electrode materials, in particular, have contributed significantly to improving the performance and miniaturization of the devices.^[^ ^22^, ^23^, ^24^, ^25^, ^26^, ^27^, ^28^, ^29^, ^30^, ^31^ ^]^ Also, different methods have been used to synthesize the materials for the preparation of the electrodes for SCs, including physical and chemical methods.^[^ ^7^, ^16^, ^18^, ^21^, ^24^ ^]^

The final device fabrication is based upon the configuration of the electrodes. If the electrodes exhibit similarity (same materials as of anode and cathode), the configuration is denoted as symmetric SCs; otherwise, it is categorized as asymmetric.^[^ ^32^, ^33^ ^]^ Several additional materials are employed to complete the device, such as wiring, packaging, and sealing, which are explained in subsequent sections.

The development of a self‐powered sensing system typically follows the ensuing steps: 1) the development of μSC arrays with high energy and power density; 2) the integration of μSCs with the sensing system, whether a sensor or biosensor; and 3) the incorporation of the SC with the energy harvesting system to facilitate its charging processes.

This work summarizes the recent advances in μSCs and their integration with electrochemical sensors. After a brief overview of SCs classification and their working principles, techniques employed for preparing μSCs and challenges related to their integration with biosensors are highlighted. Various types of SCs and technology of different self‐power‐based sensors are also considered.

## 2 Classification and Working Principles of SCs

**Figure 1:**
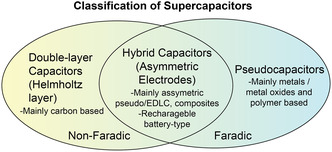
Classification of SCs showing the difference between double‐layer capacitors, hybrid capacitors, and pseudocapacitors based on the specific charge storage mechanisms.

SCs are classified into three types^[^ ^7^, ^23^, ^24^ ^]^: pseudocapacitors, electric double‐layer capacitors (EDLCs), and hybrid capacitors (**Figure** **1**).^[^ ^24^, ^34^, ^35^ ^]^ Each class is characterized by a specific charge storage mechanism, which is of type: Faradaic, non‐Faradaic, and a combination of the two, respectively.^[^ ^35^ ^]^

**Figure 2:**
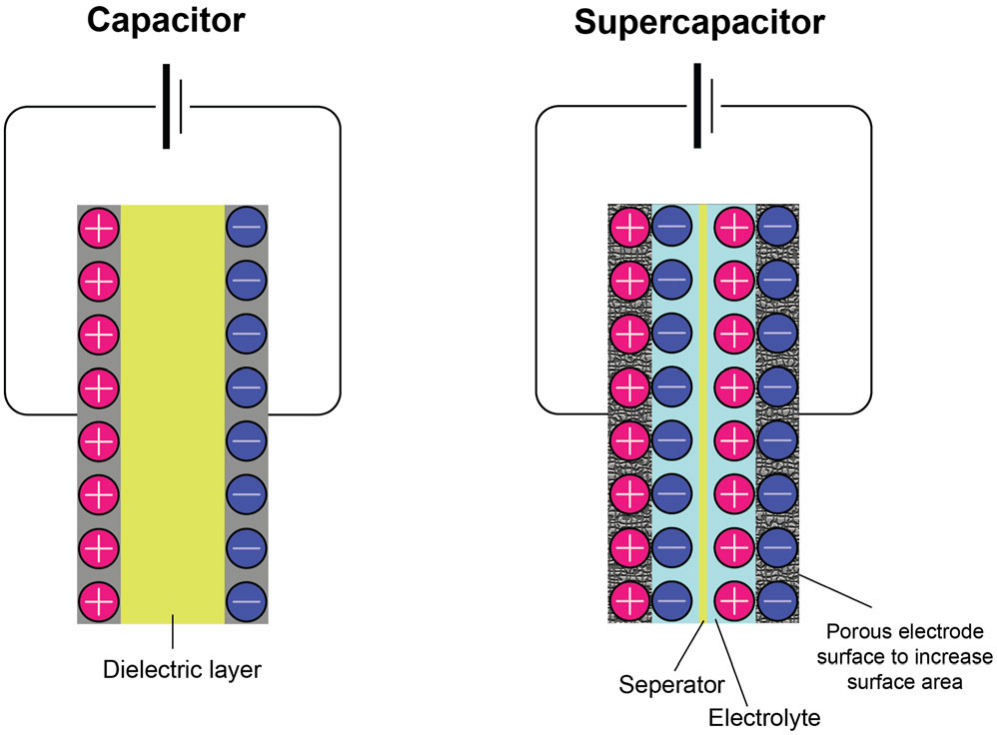
The difference in charge storage mechanism between capacitors and SCs, respectively.

In the Faradaic SCs, when a potential is applied to the two electrodes, the charge is transferred at the interface between the electrode and electrolyte due to a redox process involving electroactive species. This, for instance, is the case shown in **Figure** **2**, where the redox couple is formed by an oxidized metal ion, M^n+^, and its reduced metallic form, M. This process is analogous to the charge/discharge process in batteries.^[^ ^36^, ^37^, ^38^ ^]^ More generally, the faradaic processes at the electrodes can involve reversible adsorption (e.g., H ions on the surface of platinum electrodes), redox reactions of transition metal oxides, and reversible electrochemical doping–dedoping processes.^[^ ^39^ ^]^

**Figure 3:**
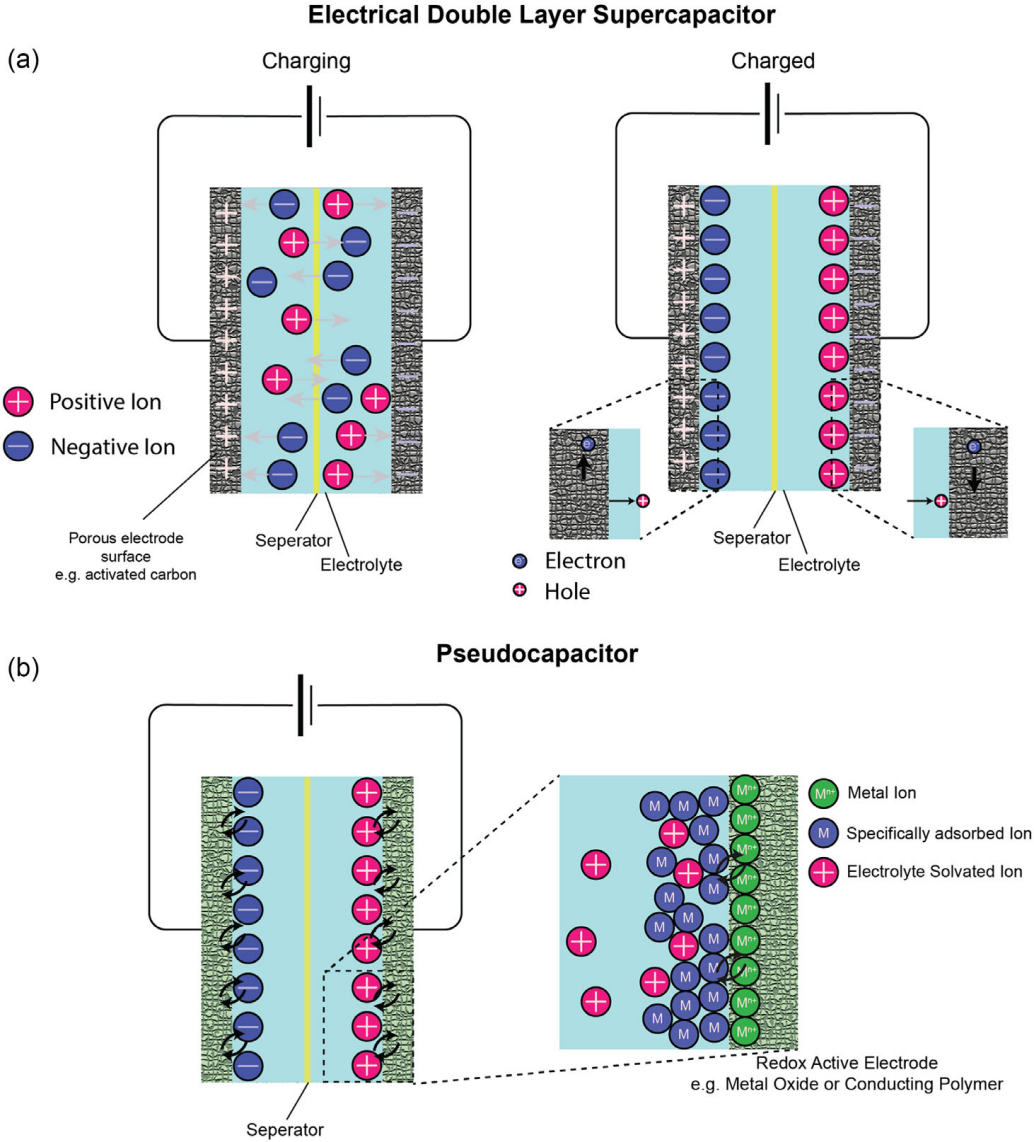
The charge storage mechanisms of the EDLC and the pseudocapacitor. a) The charging processes of the EDLC. During the charging process, negative charges migrate from the negative terminal to the positive terminal, while positive charges move vice versa. The EDLC is considered charged once saturation is achieved. b) The storage mechanism of the pseudocapacitor, wherein both electrostatics (like in EDLC) and electrochemical processes take place.

In non‐Faradaic SCs (EDLCs), charges are distributed on the surface of the electrode through physical processes, not involving any chemical reaction (e.g., breaking chemical bonds), as shown in **Figure** **3**a.^[^ ^40^, ^41^ ^]^ In particular, the ions adhering to the electrode surface form an electric double layer, the charge is stored electrostatically, and no charge transfer between electrode and electrolyte occurs. In the EDLCs, the two electrode/electrolyte interfaces of the SC are divided by a separator. In the charging phase, electrons migrate from the negative electrode to the positive electrode via the external circuit, accompanied by anions converging toward the positive electrode and cations toward the negative electrode within the electrolyte. Conversely, electrons and ions undergo reversible movement in the discharging phase. This scheme is different from that of a conventional capacitor, in which a dielectric material or nonconductive region keeps two plates or conductors apart.^[^ ^42^ ^]^ In hybrid SCs, electrostatic and electrochemical charge storage processes occur; in particular, one‐half of the hybrid SC acts as an EDLC while the other half behaves as a pseudocapacitor (Figure 3b).^[^ ^40^ ^]^ Additionally, hybrid SCs can accommodate both symmetric and asymmetric electrode configurations. SC comprises two identical electrodes in the symmetric configuration, whereas the SC comprises two dissimilar electrodes in the asymmetric configuration. Typically, hybrid SCs exploit the advantages of both types of SCs. It must be noted that while there are differences among the various SCs, the equations governing capacitance, energy density, and power density remain the same.

## 3 Electrodes and Electrolytes for SCs

Electrodes and electrolytes are critical components that affect the performance of a SC. In general, electrodes are thin, active coating material that adheres to a conductive current collector. The selection and optimization of the material to be used should consider, among others, the following features:^[^ ^16^, ^40^, ^43^ ^]^ 1) High surface area per unit volume and porosity, as they allow improving the contact between electrode and electrolyte, thus making faster the reaction kinetics. These characteristics can be attained by moving to the material at the nanoscale range and forming different morphologies. 2) Excellent electrical conductivity can enhance the energy density by decreasing the charge‐transfer impedance. 3) Better surface wettability that ensures good interaction between electrode and electrolyte. 4) Thermodynamic stability over a large potential range to achieve better cyclic stability. 5) Tunable nanostructure morphologies to increase the number of active sites, thus allowing more charges to be stored.

For EDLCs, carbon‐based materials, such as activated carbon (with surface functionalization), carbon nanotubes (CNTs), carbide‐derived carbons, and graphene, are usually employed.^[^ ^44^, ^45^ ^]^ As the EDLCs show non‐Faradaic features, their stability can be up to 10^6^ cycles or more, with a fast charge–discharge rate.^[^ ^23^, ^38^ ^]^ For pseudocapacitors, a variety of transition metal oxides, nitrides, sulfides, and conducting polymers are most commonly used.^[^ ^23^ ^]^ Promising electrode materials are Metal‐Organic Frameworks (MOFs), MXenes, and metal–organic gels, which attract significant interest because of their tunable pores and unique architectures, leading to a substantial increase in surface area.^[^ ^46^, ^47^, ^48^ ^]^ Conducting polymers, like polypyrrole (PPy), polyaniline, polythiophene, and poly(3,4‐ethylene dioxythiophene (PEDOT), are gaining importance due to their high intrinsic conductivity.^[^ ^47^, ^49^, ^50^ ^]^ Due to the continuous swelling and shrinkage of the polymer chains during the charging and discharging processes, these polymers usually exhibit low cycling stability.^[^ ^49^, ^50^ ^]^ Flexible electrode materials represent another appealing category of materials utilized in developing next‐generation portable, lightweight consumer devices, which are based on flexible SCs. These SCs can employ various mechanisms for charge storage, such as EDLCs or pseudocapacitors, contingent upon the characteristics of the materials utilized. Recently, a diverse array of flexible materials has been employed in the fabrication of flexible SCs. Examples include carbon‐based materials (such as graphene and CNTs), metal oxides (like MnO_2_ and Co(OH)_2_), MOFs, and polymer‐based materials such as poly(3,4‐ethylene dioxythiophene), polyaniline, and PEDOT:PSS, etc.^[^ ^51^ ^]^

Electrolytes notably influence the performance of SCs as they affect the voltage window and ionic conductivity.^[^ ^35^, ^41^, ^43^ ^]^ The voltage window is essential for determining energy density and primarily relies on the chemical kinetics and thermodynamic stability of the electrolyte. On the other hand, the ionic conductivity of the electrolyte impacts the power density of the SC.^[^ ^43^ ^]^ The electrolytes in the SCs can be of solid or liquid type. Liquid electrolytes include aqueous solutions of acids, bases, neutral salts, and nonaqueous solutions of organic solvents and room‐temperature ionic liquids. Solid electrolytes consist of inorganic compounds or polymer‐based membranes. Mostly, aqueous electrolytes are low cost, environmentally friendly, and have excellent ionic conductivity. The main limitation is the instability at high potentials, which decreases the capacitance of the SCs. Conversely, organic electrolytes are quite stable at high potential windows, thus enhancing the energy density performance, but they display higher resistance to ion mobility. Ionic liquids are other types of nonaqueous electrolytes that display broader stability windows, nonflammability, and higher thermal and electrochemical stability.^[^ ^41^ ^]^ Solid electrolytes are also promising alternatives, as they provide wide electrochemical stability windows, are nonflammable, and are free from leakage problems.^[^ ^43^, ^52^ ^]^ Several reviews describe the general and specific features of the various components of SCs. Readers are referred to the literature cited for more detailed accounts.^[^ ^11^, ^12^, ^41^, ^43^ ^]^

Once the various components have been selected, it is also necessary to adopt suitable technology for their assembly and for the large‐scale production of the SCs. These aspects are more challenging when devices at micrometer‐sized levels must be fabricated. To this end, several strategies have been adopted to develop μSCs and are categorized based on the materials (e.g., inks, gel electrolytes, and so on) or techniques (e.g., screen printing, laser scribing, photolithography, and so on) employed for their preparation. In the next section, we will emphasize techniques utilized to develop μSCs, which can be integrated with wearable or self‐powered sensors.

After optimizing the SC's performance with suitable electrode materials and electrolytes, the subsequent phase involves device assembly. This assembly is contingent upon electrode configuration, which broadly falls into two categories: symmetric and asymmetric SCs. Symmetric SCs employ identical electrode materials for both the anode and cathode, whereas asymmetric ones utilize distinct materials for each electrode.^[^ ^32^ ^]^

The full device fabrication process also incorporates additional materials known as wiring, packaging, and sealing materials. Wiring materials primarily consist of conductive substances such as copper and aluminum. These materials facilitate the connection of SCs to electrical circuits, enabling the charging and discharging processes.

Packaging materials predominantly comprise 3D‐printed plastic casings. These casings serve multiple functions, including shielding the SCs from environmental contaminants such as dust and moisture, providing mechanical support, and offering electrical insulation.

Sealing materials, such as epoxy resins, silicones, and polyurethanes, are predominantly employed to safeguard the device against electrolyte leakage, thereby ensuring its long‐term functionality and integrity.^[^ ^53^, ^54^, ^55^ ^]^

## 4 Techniques for the Fabrication of μSCs

### 4.1 Ink‐Based Printing Techniques

Ink‐based printing techniques are based on the diverse methods of transferring ink onto various surfaces or substrates to produce patterns, images, text, and so on The choice of ink and printing method may depend on the specific requirements of the printing application. Typical examples of this technique involved screen printing, inkjet printing (a digital printing technique that involves the precise deposition of small droplets of ink onto a substrate), mechanical scribing, and so on

Ink‐based printing techniques are used for large‐scale μSCs fabrication. Developing fully printed μSCs necessitates formulating all functional materials into printable inks, especially for electrode preparation. To simultaneously fulfill the requirements for preparing printable electrodes, inks should comprise three components: active nanoparticles or microparticles, inactive organic binders/additives, and solvents.^[^ ^56^, ^57^, ^58^ ^]^

**Figure 4:**
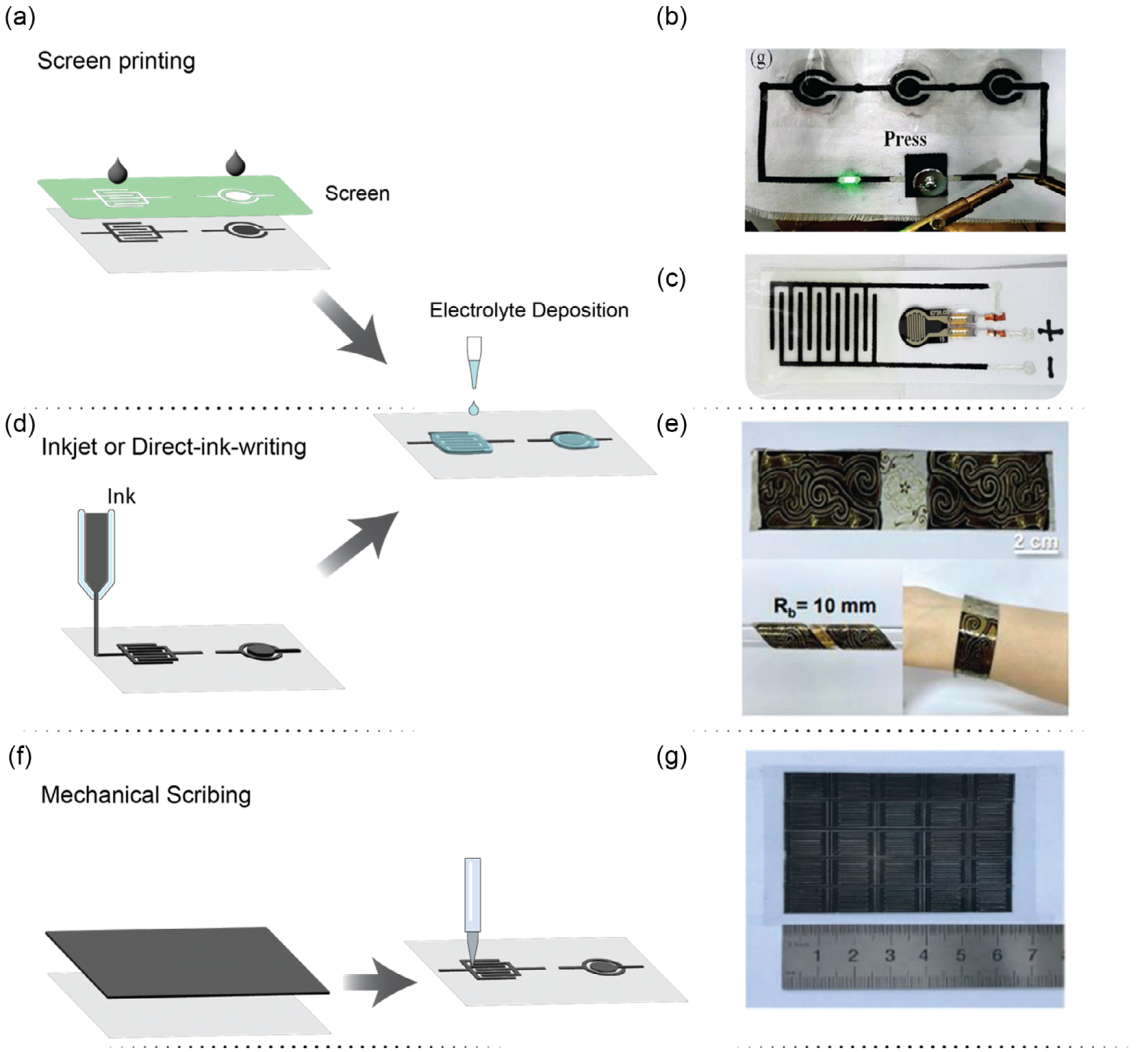
a) The ink‐based screen‐printing technique. b) The schematic illustration of the array of μSCs integrated with the pressure sensor developed by the screen‐printing technique. Reproduced with permission.^[^ ^63^ ^]^ Copyright 2022, Elsevier. c) The schematic illustration of the μSC‐integrated force sensor developed by screen printing technique. Reproduced with permission.^[^ ^64^ ^]^ Copyright 2018, Elsevier. d) The illustration of inkjet or direct ink writing. e) The Mxene‐based μSCs fabricated by inkjet or direct ink writing technique integrated with force sensor. Reproduced with permission.^[^ ^66^ ^]^ Copyright 2022, Elsevier. f) The schematic illustration of the mechanical scribing method. g) The flexible band shape μSCs developed by the mechanical scribing method integrated with the temperature sensor. Reproduce with permission.^[^ ^67^ ^]^ Copyright 2018, Welly.

The electrochemical properties of the printed μSCs are mainly dependent on the dispersion state, specific surface area, and the density of the loaded active micro‐/nanoparticles.^[^ ^59^, ^60^, ^61^, ^62^ ^]^ For instance, Shi et al. prepared a μSC. Utilizing a highly stable and conductive ink involves blending high‐quality graphene with large lateral size of 5–10 μm and a high C/O ratio (>17), along with conducting carbon black and a poly(vinyl chloride*‐co*‐vinyl acetate) (PVC/VAc) binder dissolved in dimethyl mixed dibasic acid ester solvent.^[^ ^60^ ^]^ The resulting ink demonstrated exceptional shear‐thinning behavior, enabling its extrusion through screen meshes when subjected to shear force, followed by rapid solidification. A customized screen featuring patterned meshes was utilized to apply the ink onto various insulating substrates such as polyethylene terephthalate (PET), A4 paper, glass, or cloth, thus creating arrays of microelectrodes. The ink was extruded through the screen onto the substrate's surface. Following the drying process of the patterned microelectrodes, a gel electrolyte comprising polyvinyl alcohol/H_3_PO_4_ (PVA/H_3_PO_4_) was deposited onto the designated area of microelectrodes and solidified. The resulting μSCs exhibited an areal capacitance of 0.89 mF cm^−2^, an energy density of 1.81 mWh cm^−3^, and a power density of 297 mW cm^−3^. In addition, the capacitance was retained even after 10 000 charge–discharge cycles. The schematic illustration of the working principle of the screen‐printing method is shown in **Figure** **4**a.

Using a screen‐printing method, Liu et al. developed a μSC using an ink obtained, by mixing a MnO_2_ nanosheet/graphene composite and 2D black phosphorus. The latter was employed to improve the conductivity of a graphene/MnO_2_ composite and to maintain the structural stability of the μSC. The μSC provided a specific capacitance of 41.7 F g^−1^ at 0.1 A g^−1^ and 21 F g^−1^ at 1 A g^−1^. A concentric μSC, obtained with the same material integrated on a flexible thin‐film pressure sensor on paper and carbon cloth, exhibited a specific capacitance of 20.15 mF cm^−2^, good flexibility (78% after being bent for 500 cycles at 90°) (Figure 4b).^[^ ^63^ ^]^ Xu et al. employed a two‐step screen‐printing process to fabricate a flexible coplanar asymmetric microscale hybrid μSC. 2D titanium carbide MXene (Ti_3_C_2_T_*x*_) was used as the negative electrode, and Co–Al layered double hydroxide nanosheets were employed to prepare the positive electrode. The assembled coplanar, all‐solid‐state, asymmetric device displayed an energy density of 8.84 μWh cm^−2^ and exhibited excellent flexibility. It was further integrated with force sensors to detect the applied pressure variation. It was used to measure the live heartbeat waveform for real‐time practical applications. The schematic illustration of the self‐powered force sensor is shown in Figure 4c.^[^ ^64^ ^]^

Inkjet printing is another technique to develop μSCs, as it is fast, easy to use, cost‐effective, and able to prepare smooth films compared to other printing techniques. The working principle illustration is shown in Figure 4d. It is a direct, precise parting method where the electrolyte needs to be deposited, similar to screen printing technology. Using this approach, Lee et al. developed μSCs using an aqueous printable MXene/PEDOT:PSS (MP) hybrid ink. The μSCs could deliver a volumetric capacitance as high as 754 F cm^−3^ and a remarkable energy density of 9.4 mWh cm^−3^, superior to previously reported inkjet‐printed μSCs. These systems could easily be integrated with microelectronics. This possibility was demonstrated by printing a series of microelectrodes of the μSCs, the sensor, and the interconnects between them in a single step. The integration of energy devices and sensors into a single platform is regarded as the development of self‐powered systems. In such systems, sensor devices draw energy directly from the integrated energy units to sustain continuous monitoring. Lee et al. developed self‐powered integrated microsystem could monitor temperature repeatedly and display robust mechanical resilience under various bending tests while being connected to a flexible solar cell.^[^ ^65^ ^]^

Lee et al. fabricated solid‐state μSCs using computer‐assisted direct ink writing technology. Activated carbon particles served as the model electrode active material, uniformly incorporated with multiwalled CNTs (MWCNTs) to enhance the electron mobility and the mechanical flexibility of the electrodes. A nickel/gold‐plated polyimide (PI) substrate functioned as a flexible current collector for the μSCs. The pristine PI film underwent ion‐beam etching to enhance interfacial adhesion with subsequent metal layers. The resulting μSCs exhibited a high areal energy density of 61.34 μWh cm^−2^, an areal capacitance of 60.58 mF cm^−2^, and demonstrated good mechanical flexibility under harsh deformation modes. Moreover, these μSCs were integrated with complexly shaped electronic devices such as humidity sensor‐integrated smart pots and temperature sensors smart armbands, underlining their potential user‐customized applications as monolithic micropower sources (Figure 4e).^[^ ^66^ ^]^

Mechanical scribing is another way to fabricate μSCs using proper inks. It is facile, cost‐effective, and able to produce μSCs devices at a large scale; the schematic illustration of the working principle of this technique is shown in Figure 4f.^[^ ^67^, ^68^ ^]^ By this approach, Huang et al. engineered flexible all‐solid‐state on‐chip μSCs arrays featuring ZnCo_2_O_4_ nanowires as interdigitated electrodes, PVA/KOH as the solid‐state electrolyte, and Ag nanowires as the current collector. The electrode ink was formulated by blending ZnCo_2_O_4_ nanowires with polyvinylidene fluoride as the solvent. For the collector, a conductive Ag nanowires ink was spin‐coated onto a cleaned PET substrate. Subsequently, a gel electrolyte of PVA/KOH was applied to the electrodes to produce the final all‐solid‐state μSCs on the chip. This method holds promise for the scalable development of large‐scale μSCs arrays. Figure 4g.^[^ ^67^ ^]^

### 4.2 Photolithography

**Figure 5:**
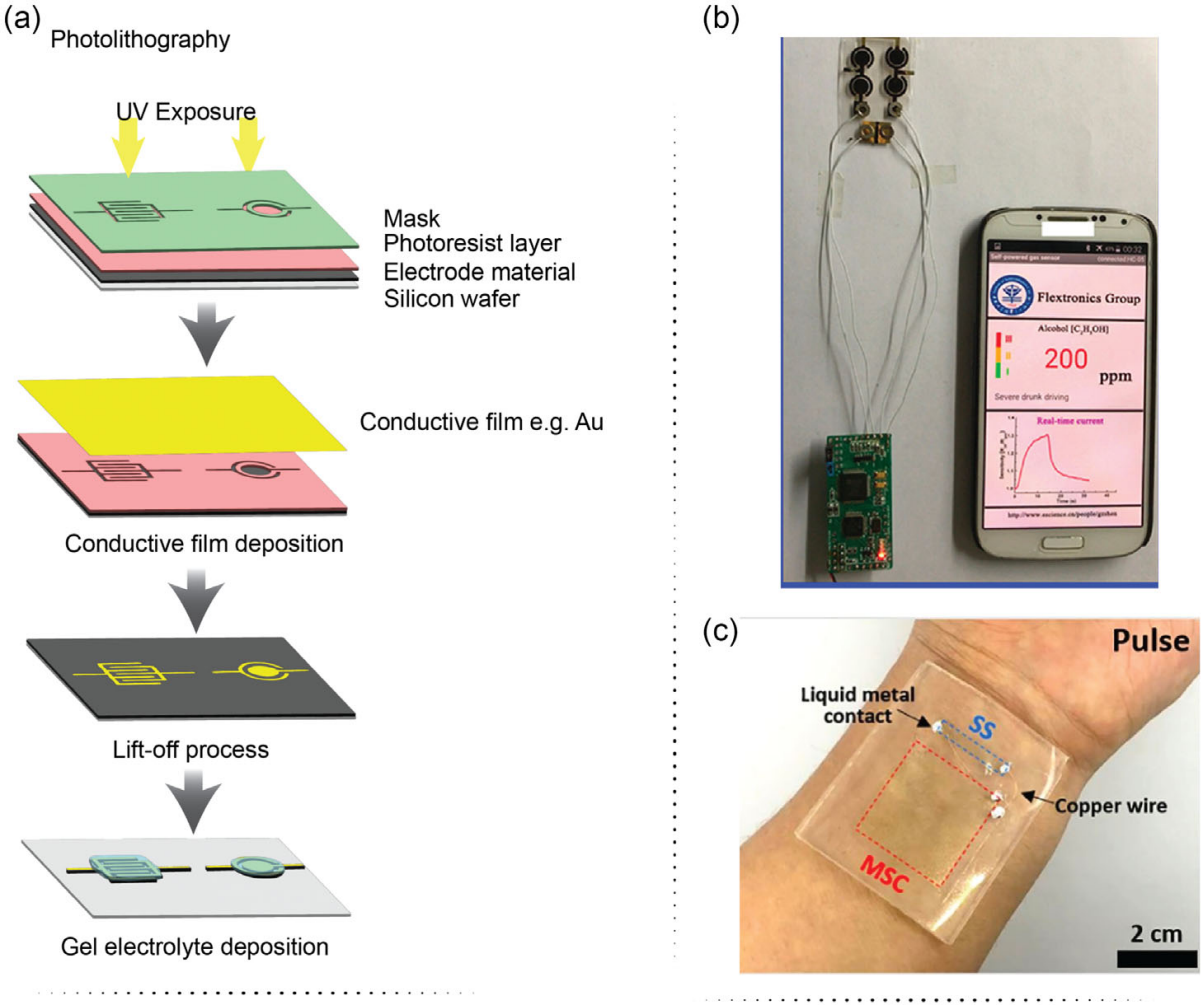
a) The step‐by‐step working principle of a photolithography technique. b) The wearable μSCs array integrated with a gas sensor. Reproduce with permission.^[^ ^75^ ^]^ Copyright 2017, Elsevier. c) The transparent stretchable μSCs integrated with a strain sensor. Reproduce with permission.^[^ ^76^ ^]^ Copyright 2022, Elsevier.

Photolithography is a technique used in the manufacturing of ICs.^[^ ^69^, ^70^, ^71^, ^72^, ^73^ ^]^ In this technique, light is used to produce patterned thin films of polymers on different substrates.^[^ ^72^, ^73^ ^]^ Usually, UV light is used to transfer a design from an optical mask to a light‐sensitive polymer photoresist coated on the substrate.^[^ ^70^, ^71^ ^]^ The wavelength of light used governs the minimum size and shape of the components integrated into the device. The technique allows the fabrication of metal conductive electrodes with high resolution, which is a useful characteristic for the development of μSCs.^[^ ^70^, ^71^, ^74^ ^]^ The step‐by‐step method of how the photolithography technique works is shown in **Figure** **5**a.

In a typical example, Li et al. developed a flexible planar concentric circular μSCs array to power gas sensors. The μSCs were obtained by combining photolithography and electrodeposition methods. First, a photolithographic process was used to pattern gold electrodes with concentric circle structures on the flexible substrate. A PPy film was electrodeposited on the concentric circle electrodes, providing highly flexible μSCs. The designed μSCs exhibited a large areal capacitance of 47.42 mF cm^−2^ with a power density of 0.185 mW cm^−2^ at an energy density of 4 μWh cm^−2^. The μSCs were further integrated to power a gas sensor (Figure 5b).^[^ ^75^ ^]^ In another work, Yun et al. developed a fractal‐designed stretchable μSC. It consisted of planar MnO_2_/CNT electrodes and 1‐butyl‐3‐methylimidazolium bis(trifluoromethylsulfonyl)imide/poly(methyl methacrylate) as an ionic electrolyte. The fractal design was obtained by an etching process based on photolithography. The fabricated μSC exhibited high transparency of 79% and a capacitance of 12.6 mF cm^−2^ at a current density of 5 mA cm^−2^. The μSC showed stable electrochemical performance even after 2000 repeated stretching cycles. The μSC was integrated into a strain sensor to obtain a single stretchable substrate. The strain sensor driven by the μSC could detect wrist pulses and bending of the wrist. The strain sensor‐integrated μSCs device represents, therefore, a useful integrated energy storage system for driving a skin‐attachable biosensor (Figure 5c).^[^ ^76^ ^]^

### 4.3 Laser Scribing

**Figure 6:**
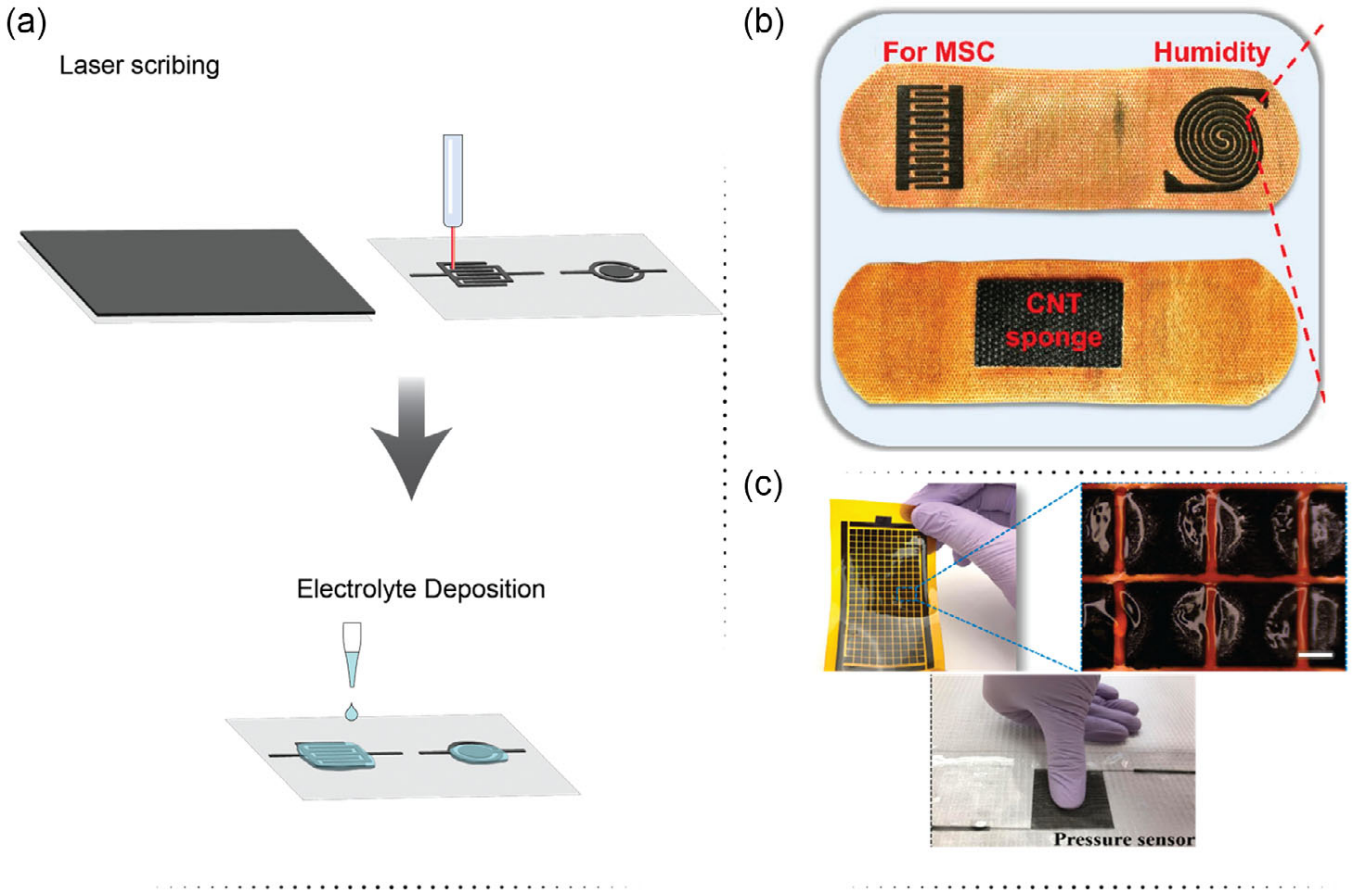
a) The working principle of laser‐scribing technique for the development of μSCs. b) The schematic illustration of μSC array on and band‐aid integrated with humidity and pressure sensor. Reproduce with permission.^[^ ^79^ ^]^ Copyright 2023, Elsevier. c) The flexible planar μSC integrated with a piezoresistive pressure sensor. Reproduce with permission.^[^ ^80^ ^]^ Copyright 2018, American Chemical Society.

Laser scribing is the process of using a laser beam to make precise cuts or grooves on a surface of materials or substrates. It is a rapid, noncontact, and single‐step technique for the fabrication of μSCs devices. This method does not require any masking, postprocessing, or complex experimental conditions (e.g., cleanrooms). It is compatible with electronic product lines for commercial use and has the potential for the preparation of self‐powered integrated devices,^[^ ^77^ ^]^ including μSCs.^[^ ^73^, ^78^ ^]^ A variety of nanomaterials, especially based on graphene, have been used to fabricate μSCs by this technique. Moreover, laser scribing technology has the advantages of direct writing and patterning the conductive electrodes into desirable shapes without any wet chemistry. This technique can, therefore, potentially be used to produce μSCs and other functional systems integrated into the same device. The working principle of laser scribing is presented in **Figure** **6**a. In a specific example, Chen et al. designed a wearable band‐aid, which integrated a μSC with humidity and pressure sensors (Figure 6b).^[^ ^79^ ^]^ The multifunctional smart system was fabricated by facile laser scribing and drop‐casting strategies. For the preparation of the μSCs, a green sodium lignosulfonate slurry was used to coat the woven fabric of the band‐aid. This step was followed by a fast laser scribing process to obtain laser‐induced sulfur‐doped porous graphene (LISG) interdigitated electrodes. The μSCs offered an outstanding capacitance of 68.6 mF cm^−2^. For the humidity sensor, graphene oxide was deposited on another engraved LISG spiral electrode. The empowered humidity sensor showed a fast response over the relative humidity (RH) sensing range of 11–97% RH. The pressure sensor was produced on the back of the band‐aid. The cotton pad was incorporated with highly conductive CNT materials and assembled with a PVA/glycerol dielectric layer film. The assembly produced a capacitive pressure sensor with a sensitivity of ≈25.15 k Pa^−1^ and a wide sensing range (2.92 Pa–300 kPa).

Taking advantage of the laser scribing technology, Lin et al.^[^ ^80^ ^]^ developed μSCs based on laser‐induced graphene (LIG) via direct laser engraving of a PI film. A CO_2_ laser (wavelength, 10.6 μm), with optimized scan rate and output power, was used to heat a Kapton film with designed 210 squares (Figure 6c). After the laser‐heating process, the gaps were brush‐coated with an electrolyte, leaving approximately 40% of each LIG square electrolyte‐free, as it happens for common conductive electrodes for in‐series connection. The electrolyte consisted of an H_2_SO_4_–PVA medium. In‐series structures of μSCs with an output voltage ranging from 1 to 6 V were fabricated. Two symmetric electrodes separated by a gap were denoted as one unit of a μSC. The three different devices (1, 3, and 6 V) showed capacitances of 58.1, 20.4, and 11.3 μF, respectively, under an applied current of 1.0 μA. It was also verified that after 5000‐cycle charge–discharge tests, the 6 V μSC retained about 97.8% of the initial capacitance. The single‐unit μSCs were also connected in series on a single‐film substrate and achieved a capacitance of 0.43 μF at a low applied current of 0.2 μA and a capacitance of 0.18 μF even at an applied current of 5.0 μA. The high‐voltage μSC was flexible with negligible degradation of charge and discharge curves at 2.0 μA when it was bent at different angles ranging from 0 to 180°. The array of 6 V μSCs was integrated into a device to power a piezoresistive microsensor to track walking activities through a wearable step counter system. Besides, a walking robot was also successfully powered by a μSCs stack for 11 s.

Another multifunctional device was developed by Mousavi et al.^[^ ^81^ ^]^ The integrated system comprised a parallel plate μSC, a humidity sensor connected in series with a resistor for data processing, and a rectangular spiral pattern serving as a near‐field communication (NFC) antenna. Laser‐scribed graphene (LSG) was utilized as the common electrode material for all components. The substrate‐free SC was produced through laser reduction of graphene oxide into graphene, followed by PANi electrodeposition. LSG films were then coated onto an ion‐porous Celgard membrane, which functioned as both substrate and separator. The resulting LSG‐PANi||LSG‐PANi SC demonstrated a volumetric capacitance of 4.6 F cm^−3^, an energy density of 0.407 mWh cm^−3^, and a power density of 196 mW cm^−3^. The humidity sensor comprised a pair of LSG electrodes with ten interdigitated fingers. To adjust the sensor's dynamic range, the area between the electrodes was printed with different grayscales. The LSG‐based device exhibited flexibility while maintaining initial performance even at a bending angle of 120° and demonstrated stability after 10 000 galvanostatic charge–discharge cycles at a current density of 833 mA cm^−3^. The built‐in humidity sensor showed a sensitivity of 15 kΩ per %RH, while the NFC module facilitated easy data transmission to a mobile phone for further processing.

### 4.4 Energy Harvester Integration

**Figure 7:**
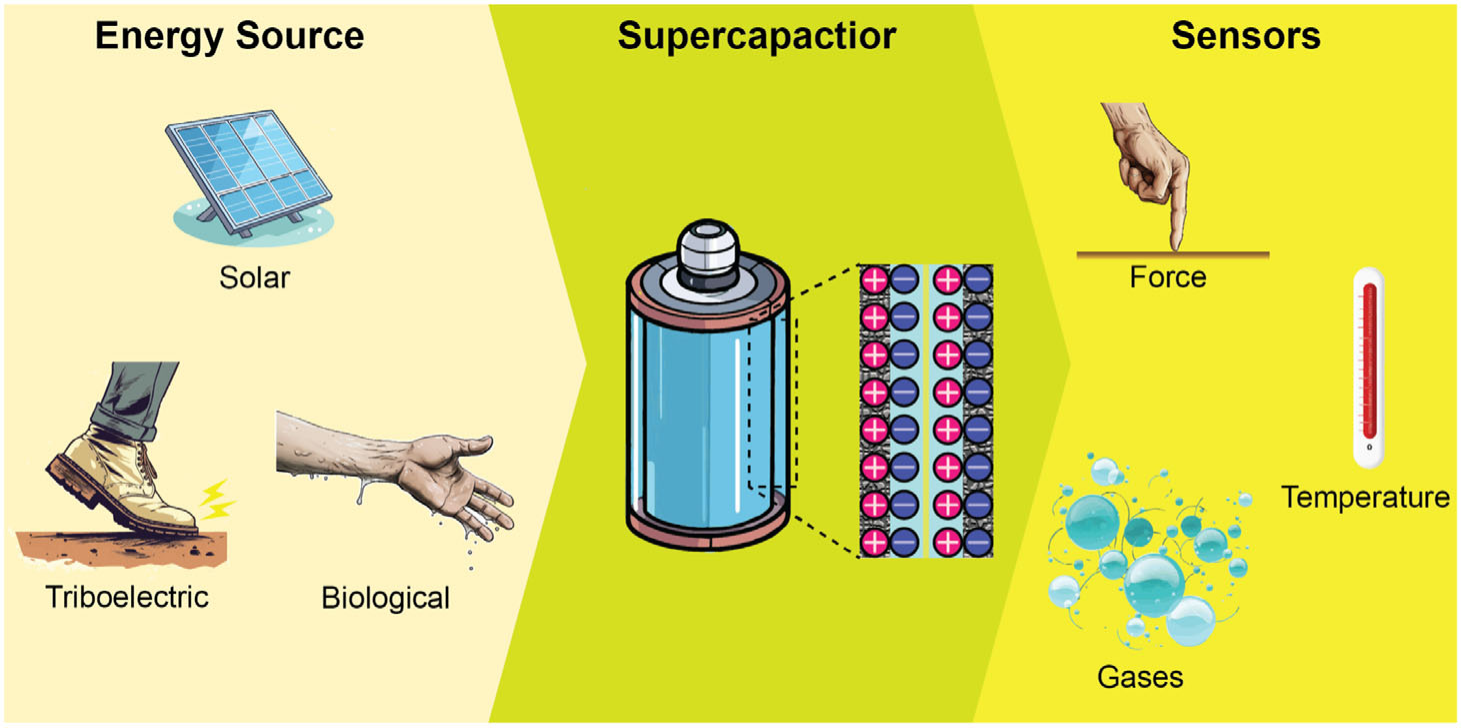
Different components (energy source, SC, and sensing system) to achieve a self‐powered sensing system.

For smart wearable monitoring systems, the ability to operate in a sustainable, independent, and maintenance‐free manner and to keep mechanical flexibility are important characteristics. As a result, several strategies have been adopted to obtain wearable, self‐powered sensing systems with integrated energy units. To this end, energy harvesters coupled to μSCs are attractive for the fabrication of self‐powered sensors for continuous monitoring in long‐term operation. Harvester systems can exploit a variety of phenomena. For instance, triboelectric nanogenerator (TENG) technology represents a significant advance in converting various mechanical energy taken from the environment into electrical energy.^[^ ^82^, ^83^ ^]^ Similarly, a piezoelectric nanogenerator generates electricity through the piezoelectric effect. It operates on the principle that when mechanical stress is applied to materials (such as bending, stretching, or any form of deformation), they generate an electric charge in response to the stress, thereby producing electrical energy.^[^ ^84^, ^85^ ^]^ Solar cells can exploit the known phenomenon of conversion of solar energy into electrical energy. On the other hand, biofuel cells (BFCs) can also be employed to exploit the oxidation of biological molecules (even from body fluids) to produce suitable electrical energy. These harvesting technologies integrated with storage systems (μSCs) could provide uninterrupted power to the monitoring systems. Some typical examples of various possible combinations among energy harvesters, SCs, and sensors, reported in the recent literature, are provided below. Several energy harvesting systems, storage systems, and sensor units to integrate and develop a self‐powered sensing system are presented in **Figure** **7**.

Several energy harvesters have been employed to develop self‐powered sensing systems in the literature. In a typical example, Zhang et al. developed a self‐powered smart sensor system based on MXene/black phosphorus by integrating a flexible pressure sensor and a solar cell with a direct laser scribing μSC to drive the operation of sensors and compensate for the intermittency of light illumination. MXene/BP lamellar structure resulted in an enhanced energy‐storage capacity of the μSC to supply enough energy for the operation of the sensor. The sensitivity of the sensor‐integrated μSC device was 77.61 kPa^−1^ at an elastic modulus of 0.45 MPa. The sensor system had response time of 10.9 ms with real‐time detection for the human heart state at physiological conditions.^[^ ^86^ ^]^ In another study, Sun et al. developed a nitrogen‐doped and carbon‐coated NiCoO_2_ (CF@NiCoO2@N–C)‐based dual‐functional electrode for simultaneous application in wearable SCs and enzyme‐free glucose sensors. The (CF@NiCoO_2_@N–C) was obtained using the electrochemical deposition method. CF@NiCoO_2_@N–C showed a specific capacitance of 644 mF cm^−2^ at 2 mA cm^−2^ and a long stable cycling performance (retaining 94% even after 10 000 cycles). In addition, the electrode material provided prominent electrocatalytic activity toward glucose. A linear relationship between current density and glucose concentration was obtained, with a sensitivity of 592 μA mm ^−1^ and a detection limit of 34.8 μm. The SC and the glucose sensor were integrated with flexible solar cells and Bluetooth into a single wearable self‐powered smart sensor system.^[^ ^87^ ^]^

Rajendran et al. pioneered the development of screen‐printed flexible and stretchable μSCs designed to serve as energy buffering components for powering wearable fitness monitoring devices. These μSCs feature interdigitated‐shaped electrodes that are inherently stretchable, along with free‐standing serpentine interconnects. They utilize CNT and poly(aniline) as active materials, while polyurethane serves as a durable binder for enduring stretch. In‐plane μSCs were initially printed on a paper substrate, precoated with a water‐soluble layer, and subsequently transferred onto a prestrained elastomeric substrate. Despite undergoing intense deformation, the μSCs exhibited remarkable mechanical resilience. Areal capacitance was 167 mF cm^−2^ at a current density of 0.4 mA cm^−2^, while areal energy and power density were 14.9 μWh cm^−2^ and 0.29 mW cm^−2^. In order to build a self‐charged and self‐powered energy storage‐harvesting hybrid device, the μSC was integrated with an amorphous silicon membrane‐based flexible solar cell (0.3 W, 2 V) and an Arduino‐based pulse rate sensor. The hybrid system efficiently powered the pulse rate monitor, and the real‐time heart rate signals were displayed continuously during exercise activities. The μSC characteristics allowed the powering of the wearable pulse rate sensor even in the presence of poor sunlight intensity.^[^ ^88^ ^]^ In a recent article, Zhang et al. introduced a cost‐effective, scalable, and straightforward manufacturing method based on LIG foams to develop a self‐powered wireless sensing platform. The 3D porous foams, characterized by their high specific surface area and enhanced charge transport, facilitated the efficient flow of triboelectric electrons in TENGs. Surface‐coated or doped foams could also create 3D composites, enhancing the energy density in arrays of μSCs. The integration of TENG and μSCs arrays efficiently harvested intermittent mechanical energy from body movements, generating stable power outputs. By patterning 3D foams and their composites into various geometries, diverse deformable sensors could be produced on a large scale at a low cost. The resulting stable yet high‐powered outputs, featuring adjustable voltage and current, enabled the operation of various stretchable sensors and wireless transmission modules. These modules wirelessly measured clinically relevant biophysical and biochemical signals, including pulse, strain, temperature, electrocardiogram, blood pressure, and blood oxygen levels. This paves the way for the early diagnostics of disease and healthy aging.^[^ ^89^ ^]^

In ref. [90], a low‐cost and scalable continuous centrifugal coating strategy was introduced for constructing in‐plane microscale self‐powered integrated systems. These devices comprise Si‐based photovoltaics as energy harvesters for electricity generation, patterned reduced graphene oxide (rGO)–CNT μSCs, and dual‐channel gas sensors for fast‐response and highly selective detection of ammonia and aniline. The gas sensors utilized PPy@rGO for NH_3_ and In_2_O_3_@rGO for aniline detection. Remarkably, the prepared high‐conducting graphene–CNT films served as both the patterned microelectrodes of embedded μSCs and the metal‐free interconnects of the circuit, providing the self‐powered systems with high integrity and flexibility. The μSCs offered a volumetric capacitance of 16.1 F cm^−3^ and a volumetric energy density of 1.43 mWh cm^−3^. The self‐powered gas detection device demonstrated a good response (≈20%, 100 ppm), linear sensitivity (from 25 to 100 ppm), and selectivity to NH_3_ and aniline.

Other approaches employed for the construction of self‐powered sensors were based on the use of biocapacitors (BCs). As an example, Lee et al.^[^ ^91^ ^]^ developed a direct electron transfer type enzyme‐based miniaturized self‐powered glucose sensor based on the BC principle, using a microsized enzyme anode of 0.1 mm^2^ surface area. To create a microscale enzyme anode capable of producing adequate power for a BC circuit, several enzyme layers consisting of flavin adenine dinucleotide‐dependent glucose dehydrogenase derived from *Burkholderia cepacia*, along with carbon black, were employed. The obtained device could detect glucose over the concentration range of 13–100 mm, based on the frequency of charge/discharge cycles of the BC. The biosensor could operate continuously for 6.6 h at 37 °C in 100 mm potassium phosphate buffer (pH 7.0). An alternative approach was employed by Bolella et al.^[^ ^92^ ^]^ to obtain a higher sensitivity and stable self‐powered biosensor to detect D‐fructose. To this end, a self‐charging biosupercapacitor (BSC) was prepared using fructose dehydrogenase as the biocomponent on the anode and laccase on the cathode. D‐fructose was used as a fuel to store the charge needed to power the sensor. The proposed BSC showed an instantaneous power density release of 17.6 and 3.8 mW cm^−2^ in pulse mode and at constant load, respectively. This platform can potentially be employed as a self‐powered biosensor in food or biomedical applications. In another study, Hou et al.^[^ ^93^ ^]^ developed a self‐powered biosensing platform that combines an enzymatic BFC (EBFC) with a corresponding capacitor for miRNA detection. They utilized a catalytic hairpin assembly and hybrid chain reaction to enhance the analytical capabilities of the EBFC. Additionally, the matching capacitor was chosen as an auxiliary device for signal amplification, and graphdiyne (2D carbon allotropes of graphene with honeycomb structures) was employed as substrate material for the construction of the EBFC. In this way, the output current of EBFC was greatly increased, providing a sensitivity of 2.75 μA pm ^−1^. MiRNA could be detected in an expanded linear range of 0.1–10^5^ fm with a detection limit of 0.034 fm. In a similar study, Wang et al. matched a capacitor to a self‐powered electrochemical biosensor for ultrasensitive determination of microRNA‐21.^[^ ^94^ ^]^ In particular, EBFCs were integrated into a capacitor‐joined circuit, and the capacitor was automatically shorted by a switching regulator to provide an instantaneous current that was rapidly detected with a digital multimeter. A sensitivity of 38.72 μA pm ^−1^ was achieved. Moreover, the redox reaction on the anode led to high‐voltage outputs at low substrate concentrations and generated electrons when the target miRNA triggered a catalytic hairpin assembly cycle. On the other hand, on the cathode, the reduction process of [Fe(CN)_6_]^3−^ was catalyzed to obtain a higher detection signal. As a result, the limit of detection of micro RNA‐21 with the developed biosensor was 0.18 fm with a linear range of 0.5–10^4^ fm.

In another report, an e‐textile microgrid system was developed by Wang et al.^[^ ^95^ ^]^ The e‐textile microgrid system integrates BFCs and triboelectric generators (TEGs), two harvesters employing distinct yet complementary energy conversion mechanisms based on user movement. It also incorporates SC modules to regulate power for wearable applications with varying power demands. During energy harvesting from human movements, TEG storage modules are activated first, leveraging instant motion‐induced charge generation to power up the system quickly. Subsequently, BFCs harvest biochemical energy from electroenzymatic reactions of sweat metabolites, enabling sustained power delivery. Additionally, a textile‐based potentiometric Na^+^ ions sensor integrated with a wearable, flexible electrochromic display (ECD) was developed as an example for applications with higher power demands operating in pulsed mode. The potentiometric sensor demonstrated a near‐Nernstian response to Na^+^ concentration, with a potential change of 57.19 mV per decade of concentration change. The sensor output could be instantly read by the preprogrammed IC, altering the color of individually controlled ECD pixels to report the data.

A variety of other self‐powered sensors and biosensors integrated with μSCs have recently been proposed. Unfortunately, for most of them, the strategies adopted were not suitable for large‐scale production. Nonetheless, it is worth considering the working principle of the various devices, the materials employed to construct both electrodes for the μSCs and electrochemical sensing systems, in view of their potential exploitation for the development of methodologies for the rapid manufacturing of all‐in‐one self‐powered devices. **Table** **1** shows a more comprehensive list of self‐powered sensors integrated with μSCs developed over the last 3 years.

**Table 1:** Self‐powered biosensors integrated with SCs system.

Power sourcea)	Energy harvester	Targeted analyte	Electrode materials for μSCs	References
μSC	External power supply	Caffeine and vanillin detection	Tungsten disulfide/PPy	[98]
Coaxial fiber‐based SC	External power supply	Strain sensing	Manganese dioxide and PPy‐deposited CNTs	[99]
SC	External power supply	Nutrition monitoring	Polyaniline/reduced graphene oxide	[100]
Textile‐based SC	External power supply	Strain sensing	MWCNT/MoO_3_ nanocomposite	[101]
Solid‐state SC	–	Strain sensing	Poly(vinyl alcohol)/poly(acrylic amide‐acrylic acid)/glycerol/NaCl	[102]

a) MWCNTs, multiwalled carbon nanotubes; MoO_3_, molybdenum trioxide; NaCl, sodium chloride.

## 5 Conclusions, Challenges, and Future Perspectives

This article highlighted the importance of self‐powered biosensors. The key is to attach the μSCs to the sensor to provide the uninterrupted power for the uninterrupted monitoring by the sensors. Several attempts were made to develop μSC‐based self‐powered systems, but there is definitely room to improve and develop an uninterrupted sensing system for deep tissue monitoring inside the body.

In future applications, the direct fabrication and connection of highly integrated, flexible sensor systems to power suppliers will enable the realization of fully self‐powered electronics without external circuitry. The practical implementation of next‐generation stretchable electronics relies on the development of sustained power supplies to drive highly sensitive wearable sensors and potentially on‐skin sensors, along with wireless transmission modules.

Despite the numerous advantages of SCs, there remain typical challenges related to enhancing energy density, developing accurate models, addressing overcharging issues, and establishing appropriate standards. As highlighted in the current review, μSCs with small size, excellent flexibility, high power density, long cycle life, and safety features have become a prominent research area. These μSCs can be charged using energy harvesters such as photovoltaic and triboelectric systems, leveraging environmental phenomena for the development of self‐powered modules.

Growing concerns about environmental sustainability have spurred the development of nanomaterials for SC fabrication, starting from renewable compounds. For instance, carbon materials are favored for energy storage devices due to their cost‐effectiveness, porous structure (providing high specific surface area), good conductivity, and high physicochemical stability. Consequently, the exploration of biomass as a source for transforming into activated carbon materials offers a promising avenue for producing environmentally sustainable energy storage devices.^[^ ^96^ ^]^

Ease of processability of electrode materials and precise control of design parameters (finger width, spacing, and thickness) using micro/nanofabrication techniques enabled tunability of energy and power performance of μSCs. While the production of stretchable self‐charging power units has been demonstrated through the integration of stretchable energy harvesters, power management circuits, and energy storage units, they frequently encounter challenges related to low and unstable output power, particularly during mechanical deformation and human movements, as well as during complex and costly fabrication processes.

Wearable sensors can detect physical and physiological biological signals in a minimal/noninvasive way. Although most sensors, especially biosensors for healthcare applications, have low power consumption, the whole sensing system that realizes data extraction, analysis, transmission, and display poses relatively high requirements on the power supply. Examples allowing us to partially overcome this problem have been provided in the review. Ensuring a stable and highly efficient power output with minimal noise remains challenging due to inevitable interference from body movements, mechanical frictions, and environmental factors. To address this challenge, there is a need for innovation in system configuration and high‐throughput fabrication methods to achieve monolithic fabrication and integration of power management circuits and supporting components into wearable platforms. Given the practical significance of devices for personalized healthcare applications, innovation in new materials with desirable properties such as breathability and washability would enhance the wearing comfort of the device.

Further challenges involve either the miniaturization of electrochemical instruments or data transfer without a wire connection, which are urgently needed. To date, electrochemical workstations are commonly used in laboratories and operated by trained personnel, which can limit their application. The miniaturization of electrochemical instruments is also an imminent requirement. To achieve the above goals, integration of other novel engineering, such as skin chip technology and cloud connectivity, is needed. With the continuous development of new technologies and materials, most research on wearable self‐powered sensors focused on Bluetooth, wireless power transfer, and field communication. In addition to engineering and design considerations, wearable physical or chemical sensors encounter a dynamic working environment during on‐body operation, introducing additional complexity and uncertainty to the real‐time collection of accurate physiological and chemical information. Usually, cross‐validation of sensor responses with laboratory gold standard methods is conducted. Therefore, it is crucial for appropriate and responsible reporting of validation data and disclosure of uncertainty, not only to ensure that results from human research hold scientific and societal significance but also to prioritize the safety of participants.^[^ ^97^ ^]^

Finally, due to the lack of scalable single fabrication techniques and the single base material to fulfill all the requirements of harvesting‐storage consumption, μSCs have not been fully commercialized yet. Additionally, the energy harvester system is another obstacle in this system. There should be a system that can operate the sensor system for a longer time without the need for any external energy harvesting system. The present literature survey could suggest to research engineers and designers which research gaps are still associated with μSCs and their integration with self‐powered sensor systems.

## Conflict of Interest

The authors declare no conflict of interest.
